# Multiregional radiomics profiling from multiparametric MRI: Identifying an imaging predictor of IDH1 mutation status in glioblastoma

**DOI:** 10.1002/cam4.1863

**Published:** 2018-11-13

**Authors:** Zhi‐Cheng Li, Hongmin Bai, Qiuchang Sun, Yuanshen Zhao, Yanchun Lv, Jian Zhou, Chaofeng Liang, Yinsheng Chen, Dong Liang, Hairong Zheng

**Affiliations:** ^1^ Institute of Biomedical and Health Engineering, Shenzhen Institutes of Advanced Technology Chinese Academy of Sciences Shenzhen China; ^2^ Department of Neurosurgery Guangzhou General Hospital of Guangzhou Military Command Guangzhou China; ^3^ Department of Radiology Sun Yat‐sen University Cancer Center Guangzhou China; ^4^ Department of Neurosurgery The 3rd Affiliated Hospital of Sun Yat‐sen University Guangzhou China; ^5^ Department of Neurosurgery/Neuro‐oncology, State Key Laboratory of Oncology in South China, Collaborative Innovation Center for Cancer Medicine Sun Yat‐sen University Cancer Center Guangzhou China

**Keywords:** glioblastoma, IDH1 mutation, magnetic resonance imaging, radiomics

## Abstract

**Purpose:**

Isocitrate dehydrogenase 1 (IDH1) has been proven as a prognostic and predictive marker in glioblastoma (GBM) patients. The purpose was to preoperatively predict IDH mutation status in GBM using multiregional radiomics features from multiparametric magnetic resonance imaging (MRI).

**Methods:**

In this retrospective multicenter study, 225 patients were included. A total of 1614 multiregional features were extracted from enhancement area, non‐enhancement area, necrosis, edema, tumor core, and whole tumor in multiparametric MRI. Three multiregional radiomics models were built from tumor core, whole tumor, and all regions using an all‐relevant feature selection and a random forest classification for predicting IDH1. Four single‐region models and a model combining all‐region features with clinical factors (age, sex, and Karnofsky performance status) were also built. All models were built from a training cohort (118 patients) and tested on an independent validation cohort (107 patients).

**Results:**

Among the four single‐region radiomics models, the edema model achieved the best accuracy of 96% and the best F1‐score of 0.75 while the non‐enhancement model achieved the best area under the receiver operating characteristic curve (AUC) of 0.88 in the validation cohort. The overall performance of the tumor‐core model (accuracy 0.96, AUC 0.86 and F1‐score 0.75) and the whole‐tumor model (accuracy 0.96, AUC 0.88 and F1‐score 0.75) was slightly better than the single‐regional models. The 8‐feature all‐region radiomics model achieved an improved overall performance of an accuracy 96%, an AUC 0.90, and an F1‐score 0.78. Among all models, the model combining all‐region imaging features with age achieved the best performance of an accuracy 97%, an AUC 0.96, and an F1‐score 0.84.

**Conclusions:**

The radiomics model built with multiregional features from multiparametric MRI has the potential to preoperatively detect the IDH1 mutation status in GBM patients. The multiregional model built with all‐region features performed better than the single‐region models, while combining age with all‐region features achieved the best performance.

## 1 INTRODUCTION

Glioblastoma (GBM, WHO grade IV glioma) is the most common malignant brain tumor, characterized by genetic instability, intratumor heterogeneity, and dismal prognosis.^1^ Genomewide‐analysis has revealed that the isocitrate dehydrogenase 1 genes (IDH1) are mutated in approximately 12% of GBM.^2^ IDH1 mutations predominantly occur in secondary GBMs and younger patients but are much rarer in primary GBMs.^3^, ^4^ IDH1 mutations are associated with longer survival and are considered as an independent prognostic indicator.^5^, ^6^ GBMs with IDH1 mutation represent a distinct disease entity with a different clinical behavior and genetic characteristics. Detection of IDH1 status is required to guide personalized therapies and recommended by the World Health Organization.^7^

Currently, the detection of IDH1 mutation focuses on genetic profiling approaches, requiring biopsy or surgical resection for tissue sampling. However, biopsy‐based approach has been controversial due to its invasiveness, potential complications, and possible incomplete sampling caused by intratumor genetic heterogeneity.^8^, ^9^ Substantial assessment requires multiregion sampling of the tumor, which currently is not widely accepted in clinical practice. An emerging technique, radiomics, allows three‐dimensional characterization of the imaging phenotypes in the entire heterogeneous tumors through high‐throughput extraction of quantitative imaging features.^10^, ^11^ Meanwhile, the advancement of imaging genomics permits correlating qualitative image phenotypes with molecular data.^12^ Imaging genomics provides a unique opportunity of radiomics to detect underlying molecular properties by noninvasive and repeatable imaging‐based approaches.

Several radiomics models associated with IDH1 mutations in low‐ (grade I and II)^13^, ^14^, ^15^, ^16^ and high‐level (grade III and IV) glioma^16^, ^17^, ^18^ have been reported. Due to the multiregional and microenvironmental heterogeneity in GBM,^19^ the high prognostic and predictive value of multiregional radiomics model from multiparametric MRI has been widely recognized.^20^, ^21^, ^22^ It is a reasonable hypothesis that imaging features from multiple tumor subregions could have the best accuracy in predicting IDH1 mutation than those from single subregion. Beyond the accuracy, researchers may be more interested in exploring the underpinning of the predictive radiomics features. Building a compact radiomics model with a minimal set of imaging features related to IDH1 mutation, rather than a model with many well‐fitted features, could be more conducive to decipher mechanism underlying an imaging genomics model. However, such an interpretable radiomics model built with a minimal set of multiregional MRI features associated with IDH1 mutation in GBM is still in demand.

In this retrospective multicenter study, we aimed to develop a radiomics model with a minimal set of imaging features from multiple tumor subregions in multiparametric MRI for pretreatment prediction of IDH1 status in GBM patients.

## 2 METHODS

### 2.1 Study population

In this retrospective study, 651 patients from The Cancer Imaging Archive (TCIA) publicly‐available dataset (www.cancerimagingarchive.net) and three local institutions between January 2013 and July 2017 were analyzed. The inclusion criteria were that patients with (a) newly diagnosed histologically‐confirmed GBM and (b) pretreatment MRI including axial T1‐weighted, axial T1‐weighted Gadolinium contrast‐enhanced, axial T2‐weighted, and T2‐weighted fluid attenuation inversion recovery images (short for T1w, T1c, T2w, and FLAIR), and (c) known IDH1 genotype determined from tumor tissue and (d) known clinical data including age, sex, and Karnofsky performance status (KPS). Exclusion criteria were that (a) patients without confirmed IDH1 data and (b) MRI with motion artifact. Finally, 225 patients were included in this study, consisting of a training cohort of 118 patients and an independent validation cohort of 107 patients. The training cohort comprised 58 patients from TCIA and 60 patients from Sun Yat‐sen University Cancer Center. The validation cohort comprised 45 patients from The 3rd Affiliated Hospital of Sun Yat‐sen University and 62 patients from Guangzhou General Hospital of Guangzhou Military Command. The study was approved by the Ethics Committee of Sun Yat‐sen University Cancer Center, the Ethics Committee of The 3rd Affiliated Hospital of Sun Yat‐sen University, and the Ethics Committee of Guangzhou General Hospital of Guangzhou Military Command. Because the data in TCIA was deidentified, Institutional Review Board approval for TCIA data was not required. Informed consent for the patients in three local institutions was waved. Patient and tumor characteristics were summarized in Table 1.

**Table 1:** Patient and tumor characteristics of the study population

Characteristic	Training cohort	Validation cohort	*P*
No. of patients	118 (52.44%)	107 (47.56%)	
Sex			0.941
Female	48 (40.68%)	43 (40.19%)	
Male	70 (59.32%)	64 (59.81%)	
Age (y)			0.960
Mean (Range)	53.6 (10‐85)	53.3 (9‐80)	
≤65	94 (79.66%)	85 (79.44%)	
>65	24 (20.34%)	22 (20.56%)	
KPS			0.963
Mean	80.93	79.72	
≤70	37 (31.36%)	31 (28.97%)	
>70	81 (68.64%)	76 (71.03%)	
IDH1			0.821
Mutated	10 (8.47%)	10 (9.35%)	
Wild‐type	108 (91.53%)	97 (90.65%)	

IDH1, isocitrate dehydrogenase 1; KPS, Karnofsky performance status.

### 2.2 MR imaging

All local MR images were acquired with 1.5 and 3.0‐T MRI systems (Magnetom Verio or Trio TIM, Siemens Healthcare, Erlangen, Germany and Discovery MR 750; GE Healthcare, Milwaukee, WI, USA). The scanning sequences and parameters were: (a) T1‐weighted sequences at repetition time milliseconds, 210‐720; echo time milliseconds, 4‐20; section thickness, 2.0‐5.0 mm; (b) T1‐weighted Gadolinium contrast‐enhanced images at repetition time milliseconds, 260‐950; echo time milliseconds, 4‐20; section thickness, 2.0‐5.0 mm; (c) T2‐weighted images at repetition time milliseconds, 2137‐10 000; echo time milliseconds, 80‐140; section thickness, 3.0‐5.0 mm; (d) T2‐weighted fluid attenuation inversion recovery images at repetition time milliseconds, 6000‐11 000; echo time milliseconds, 85‐155; section thickness, 2.5‐6.0 mm.

### 2.3 IDH1 mutation analysis

For the TCIA patients, IDH1 mutation data were obtained from The Cancer Genome Atlas (TCGA) publicly‐available dataset corresponding to the TCIA patients. For patients from the three local institutions, IDH1 status was assessed by pyrosequencing approach. The DNA was isolated from paraffin sections of tumor tissue using QIAamp DNA FFPE Tissue Kit (Qiagen, Hilden, Germany). Pyrosequencing analysis was performed using the PyroMark Q96 system (Qiagen) with polymerase chain reaction (PCR) products of exon 4 of IDH1 containing R132 coding region.

### 2.4 Image preprocessing and multiregional segmentation

Image preprocessing was critical for extracting stable features and achieving reproducible results in this multicenter study where MRI data were acquired from multiple scanners. All images were preprocessed to standardize the intensity and geometric variations of the MRI data. First, a N4ITK correction was used to correct the bias field distortion.^23^ After skull stripping, all voxels were isotropically resampling into 1 × 1 × 1 mm^3^. With the ITK software (https://itk.org/), rigid body registration was performed with the mutual information similarity metric using T1c as a template. To standardize the intensity variation between MRI acquisitions across multicenter study, an efficient landmark‐based piecewise intensity mapping was used for histogram matching.^24^ Having normalized T1w, T1c, T2w, and FLAIR images, the brain was automatically segment into five classes: the non‐tumor region and four tumor subregions including necrosis, edema, non‐enhancement area, and enhancement area. The segmentation procedure was automatically accomplished by using a convolutional neural network (CNN)‐based method.^25^ To train the CNN model, real patient MR data from the Multimodal Brain Tumor Image Segmentation Benchmark (BRATS) 2017 was used.^26^ BRATS is a well‐established benchmark for training and evaluating brain tumor segmentation algorithms, providing publicly‐available standard glioma MRI datasets (T1w, T1c, T2w, and T2 FLAIR) with expert‐outlined tumor subregions (necrosis, edema, non‐enhancement area, and enhancement area).

### 2.5 Multiregion radiomics feature extraction

From the segmented subregions, high‐throughput imaging features were extracted, including location features, geometry features, intensity features, and texture features. To fully characterize the tumor heterogeneity, we extracted in total 1614 image features from multiple tumor subregions, including necrosis, enhancement area, non‐enhancement area, edema, solid core (the whole tumor except edema), and whole tumor. The radiomics features extracted were summarized in Table 2.

**Table 2:** A summary of the radiomics features extracted. Note that there were two different calculations for both GLCM Homogeneity and GLCM Informational Measure of Correlation, which can be found in ref. [27]

Feature classes	Feature names
Location features	Region (Frontal, Temporal, Insular, Parietal, Occipital, Brainstem, Cerebellum); Side (Right, Left, Bilateral)
Geometry features	Volume, Subregion Proportion, Surface area, Longest Diameter, Solidity, Eccentricity, Compactness, Spherical Disproportion, Surface Area to Volume Ratio, Sphericity
Intensity features	Max Value, Median Value, Min Value, Mean Value, Range, Energy, Entropy, Variance, Kurtosis, Uniformity, Root Mean Square, Skewness, Standard Deviation, Mean Absolute Deviation
Texture features	
GLCM features	Contrast, Correlation, Autocorrelation, Energy, Variance, Dissimilarity, Entropy, Sum Average, Sum Entropy, Sum Variance, Difference Variance, Difference Entropy, Cluster Prominence, Cluster Shade, Maximum Probability, Homogeneity 1/2, Informational Measure of Correlation 1/2, Inverse Difference Moment Normalized, Inverse Difference Normalized
GLRLM features	Short Run Emphasis, Long Run Emphasis, Gray‐Level Nonuniformity, Run‐Length Nonuniformity, Run Percentage, Low Gray‐Level Run Emphasis, High Gray‐Level Run Emphasis, Run‐Length Variance, Short Run Low Gray‐Level Emphasis, Short Run High Gray‐Level Emphasis, Gray‐Level Variance, Long Run Low Gray‐Level Emphasis, Long Run High Gray‐Level Emphasis
GLSZM features	Small Zone Emphasis, Large Zone Emphasis, Gray‐Level Nonuniformity, Zone‐Size Nonuniformity, Zone Percentage, Low Gray‐Level Zone Emphasis, High Gray‐Level Zone Emphasis, Zone‐Size Variance, Small Zone Low Gray‐Level Emphasis, Small Zone High Gray‐Level Emphasis, Gray‐level Variance, Large Zone Low Gray‐Level Emphasis, Large Zone High Gray‐Level Emphasis
NGTDM features	Coarseness, Contrast, Busyness, Complexity, Strength

GLCM, gray‐level co‐occurrence matrix; GLRLM, gray‐level run length matrix; GLSZM, gray‐level size zone matrix; NGTDM, neighborhood gray‐tone difference matrix.

The location features indicating the tumor geographic epicenter were defined by two features (region and side, as shown in Table 2) according to the VASARI guideline.^27^ The locations were determined by three neurologists (H.B. with 12‐year experience in neuroradiology; Y.C. and C.L., each with 5‐year experience in neuroradiology) and two radiologists (Y.L. and J.Z., each with more than 7‐year experience in neuroradiology). The geometry features characterized the three‐dimensional shape of tumor subregions. A total of 28 geometry features were extracted. The intensity features described the first‐order distributions of the voxel intensities within the subregions. For six extraction subregions in four MR modalities, 336 intensity features were extracted. The texture features described the high‐order distributions of the intensities. The texture features were extracted using the gray‐level co‐occurrence matrix (GLCM), gray‐level run length matrix (GLRLM), gray‐level size zone matrix (GLSZM), and neighborhood gray‐tone difference matrix (NGTDM) methods. GLCM measured image properties related to second‐order statistics (textural properties between two voxels). GLRLM reflects the distribution of gray‐levels of runs, where a gray‐level run is a set of consecutive collinear voxels having the same gray‐level value. Different from the gray‐level run, GLSZM makes use of the gray‐level size zone, which is a flat area with the same gray‐level. NGTDM reflects a gray‐level difference between voxels with certain gray‐level and their neighboring voxels. All texture features were calculated with 26‐voxel connectivity in 13 directions, where the intensities were quantized to 64 gray‐levels using a Lloyd‐Max quantization method. The detailed calculation of the texture features can be found in ref. [28]. A total of 1248 texture features were computed from six extraction subregions and four modalities. All radiomics features were extracted using an in‐house Matlab program. There are also several open‐source tools for extraction of radiomics features, such as python package pyradiomics and R package Radiomics.

### 2.6 Data balancing

Because mutated IDH1s were rare in GBM, the classes (mutated and wild‐type IDH1) were not equally represented, as shown in Table 1. This imbalance could lead to poor predictive accuracy for the minority class for most machine learning‐based classification models. Resampling was a commonly used method to address the imbalanced learning problems. Here we used the synthetic minority over‐sampling technique (SMOTE) algorithm^29^ to improve the imbalance, where more minority instances were generated along a line joining a minority instance and its selected nearest neighbors. After intensive testing, the instance number of minority class was set to 64 to achieve an optimal classification result. Particularly, 54 extra instances were generated from the minority class, while the majority class remained unchanged. Note that the data balancing was performed only on the training dataset. The R package smotefamily was used for data balancing.

### 2.7 Feature selection and classification

The feature selection was assumed to discover a minimal set of features relevant to IDH1 mutation. A random forest‐based wrapper method, named Boruta, was used for relevant feature selection.^30^ Boruta algorithm has been successfully used in genomics analysis to select genes related to cancer, as in the way here to select features relevant to IDH1 mutation. Boruta evaluated relevant features iteratively by comparing the importance of original features measured by random forest with that achieved by artificially added random features. A random forest algorithm was performed iteratively to measure the feature importance. In each iteration, if a feature achieved higher importance than the artificially added random features, it was deemed relevant. Otherwise, that feature was considered irrelevant, leading to the removal of the feature. The process was repeated to achieve statistical significance and finally generate a minimal set of the most relevant features. The R package Boruta was used to build the model.

To compare radiomics features from different tumor subregions, four single‐region models and three multiregional models were built. The four single‐region models were built based on imaging features from the enhancement area, non‐enhancement area, necrosis, and edema, respectively. The three multiregional models were built with imaging features extracted from the tumor core, the whole tumor, and all six regions (as introduced in the feature extraction subsection). Furthermore, a combined model was built based on all‐region radiomics features and clinical factors (sex, age, and KPS). When building the seven radiomics models and the combined model, Boruta was used for feature selection and random forest model was used for predicting IDH1 mutation. A clinical model based on clinical factors alone was also built using random forest. After a set of testing, the tree number of all random forest models was set to 300, as increasing the tree number did not bring significant performance gain. Gini index was used as an importance measure. The R package randomForest was used for model building.

### 2.8 Statistical analysis

The statistical analysis was done with R software, version 3.4.3 (https://www.r-project.org/). Two‐sided *P* value of <0.05 was considered significant. The differences in sex, age, KPS, and IDH1 mutation status between the training and validation data sets were assessed. According to the guideline in ref. [26], the segmentation algorithm was tested via BRATS online evaluation tool, where the segmentation performance of three regions (the whole tumor, the tumor core, and the enhancement area) was evaluated in terms of DICE score, sensitivity, and specificity. All classifiers were trained on the training cohort and tested on the independent validation cohort. The classification performance was assessed by using several indices, including accuracy (ACC), sensitivity (SEN, also referred to as recall), specificity (SPE), and precision (PRE). The overall performance was evaluated using the area under the receiver operating characteristic (ROC) curve (AUC). The maximum value of the Youden index (sensitivity + specificity−1) was used as the cutoff. The DeLong method was used for statistical comparison of AUCs.^31^ As suggested in ref. [32], for comprehensive evaluations of the classification performance on the imbalanced dataset, the precision‐recall curves (PRC) and F1‐score were calculated. The PRC, defined by plotting precision rate over the recall rate, could give a more informative picture of an algorithm's performance than conventional ROC in the presence of imbalanced data. F1‐score, defined as 2 precision∙recall/(precision + recal), provided more insight into the functionality of a classifier than the accuracy metric. All indices were calculated for both training and validation cohorts.

## 3 RESULTS

**Figure 1:**
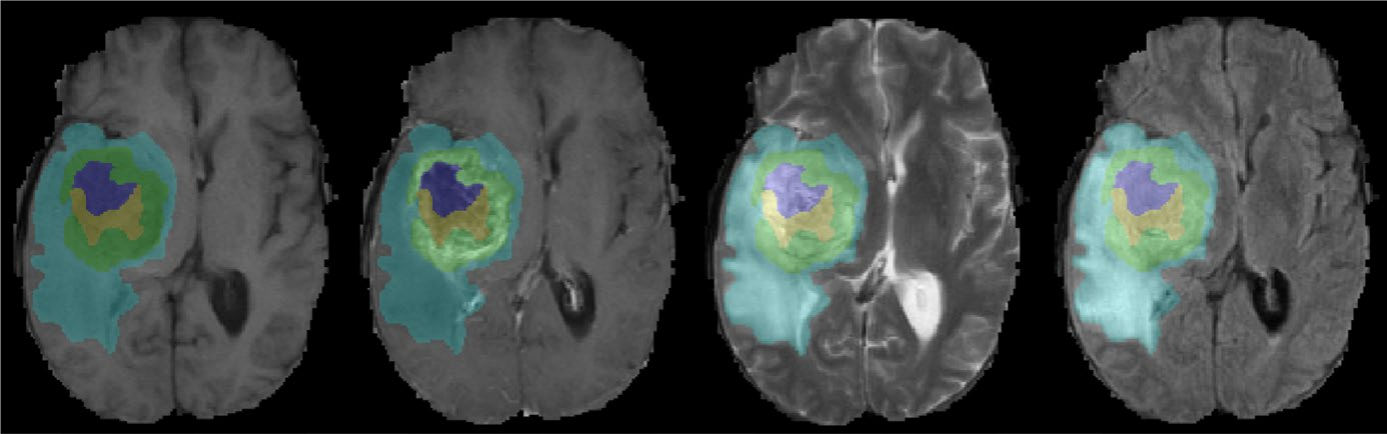
Multiregional segmentation result. The enhancement area, non‐enhancement area, necrosis, and edema were shown in green, yellow, purple, and blue, respectively

**Figure 2:**
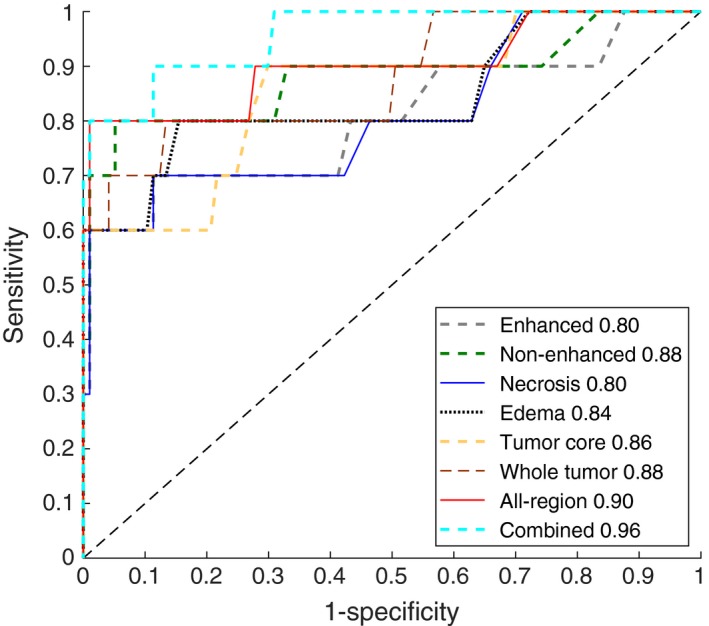
Receiver operating characteristic (ROC) curves of the multiregional and single‐region radiomics models in the independent validation cohort, where the area under the receiver operating characteristic curve (AUC) for each model was displayed

**Figure 3:**
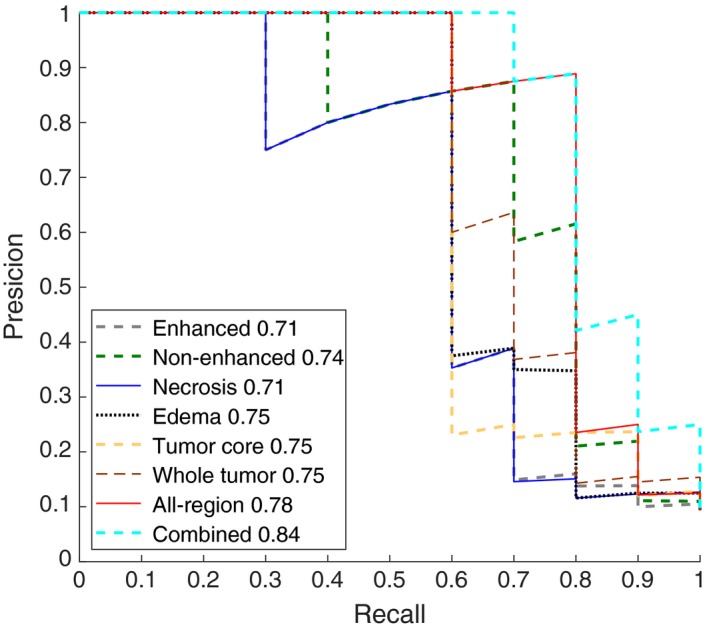
Precision‐recall curves (PRC) of the multiregional and single‐region radiomics models in the independent validation cohort, where the F1 score for each model was displayed

**Figure 4:**
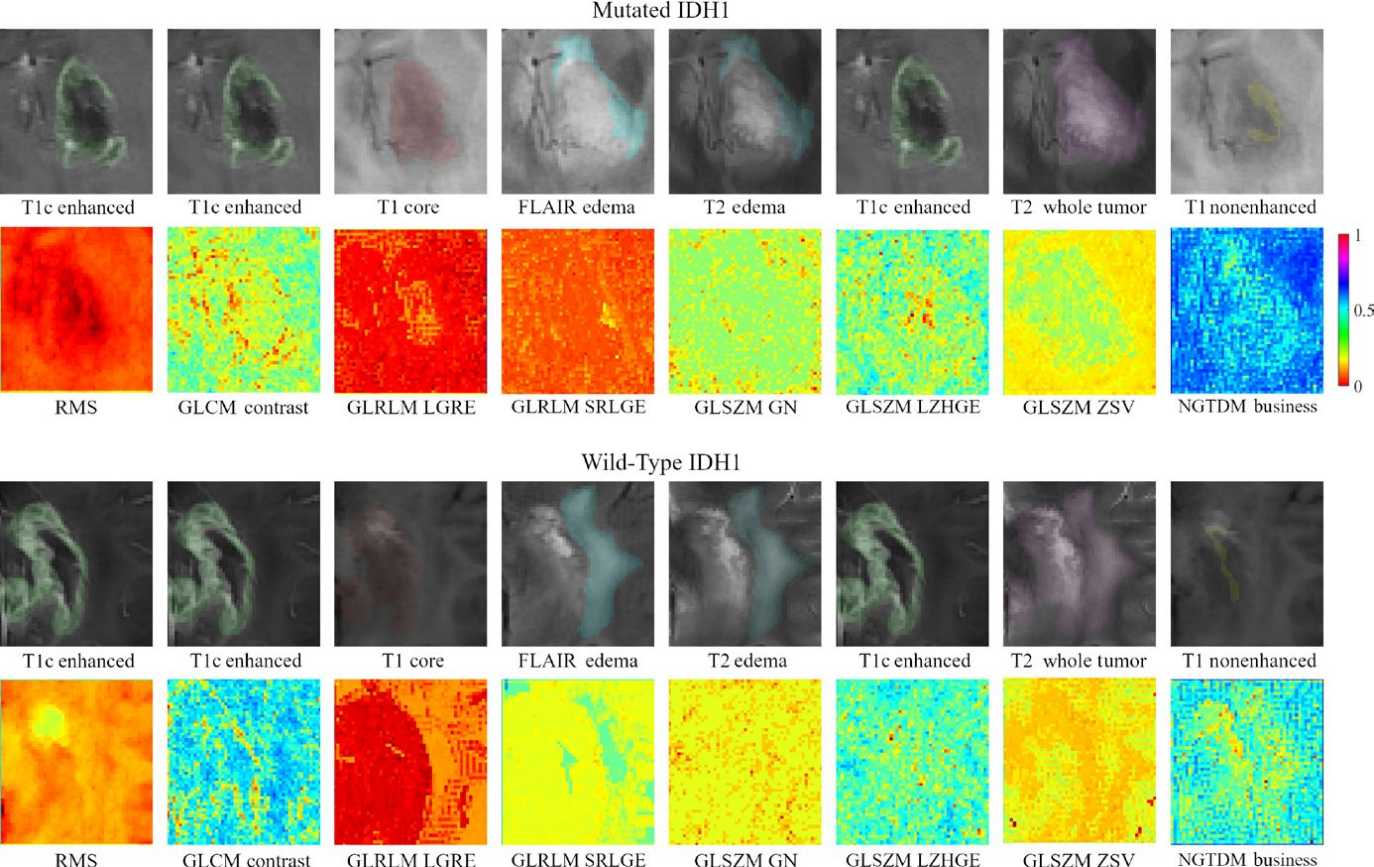
Feature maps of the eight all‐region features for an isocitrate dehydrogenase 1 (IDH1)‐mutated patient (top) and an IDH1‐wild‐type patient (bottom). The feature maps illustrated how the selected features radiologically quantified the multiregional variations. Specifically, the features *f*_1_ measured the quadratic mean of the intensity within the enhancement area; *f*_2_ measured the amount of local variations present in the enhancement area; *f*_3_ indicated the spatial distribution of low‐level intensity within core area; *f*_4_ characterized the joint distribution of both low‐level intensity and short run length within edema; *f*_5_ quantified the nonuniformity of gray‐level within edema; *f*_6_ described the distribution of both high‐level intensity and large area size within the enhancement area; *f*_7_ described the variance of the size of area with the same gray‐level in the whole tumor region; *f*_8_ described the spatial rate of intensity change within the non‐enhancement area

No difference was found between the training dataset and the validation dataset in sex, age, KPS, and IDH1 mutation status (*P *= 0.821‐0.963, as shown in Table 1). The multiregional segmentation result was shown in Figure 1. The segmentation performance was summarized in Table 3, where each measure was given as mean ± standard deviation. For the all‐region radiomics models, eight features remained after feature selection, as shown in Table 4. The features selected for building the tumor‐core model, the whole‐tumor model, and four single‐region models were shown in Table 5. For the combined models, six imaging features and age remained after feature selection, as shown in Table 5. Among four single‐region radiomics models, the model built from edema region achieved the best accuracy of 96% and the best F1‐score of 0.75, while the model built from non‐enhancement region achieved the best AUC of 0.88 in the independent validation cohort. Generally, the overall performance of the tumor‐core model (accuracy 0.96, AUC 0.86, and F1‐score 0.75) and the whole‐tumor model (accuracy 0.96, AUC 0.88, and F1‐score 0.75) was slightly better than the single‐regional models. The all‐region radiomics model achieved an improved overall performance of an accuracy 96%, an AUC 0.90, and an F1‐score 0.78 in the validation cohort. Among all models, the combined model achieved the best performance of accuracy 97%, AUC 0.96, and F1‐score 0.84 in the validation cohort. The ROC and PRC curves for three multiregional models, four single‐region models, and the combined model in the validation cohort were shown in Figures 2 and 3, respectively. The DeLong analysis found that in the validation cohort the AUC of the all‐region model was significantly higher than those of all single‐region models, while the AUC of the combined model was significant higher than those of all the other models (*P* < 0.05). To further reveal the relevance of the selected eight multiregional features with the IDH1 mutation status, the feature maps were presented in Figure 4 for an IDH1‐mutated patient and an IDH1‐wild‐type patient. The performance of all predictive models was summarized in Table 6.

**Table 3:** A summary of the segmentation performance

Tumor region	DICE score	Sensitivity	Specificity
Whole tumor	0.885 ± 0.050	0.889 ± 0.082	0.971 ± 0.012
Tumor core	0.831 ± 0.100	0.845 ± 0.066	0.988 ± 0.009
Enhancing area	0.867 ± 0.108	0.825 ± 0.117	0.989 ± 0.005

**Table 4:** A summary of the selected features used for building the all‐region model

No.	Selected feature	Type	Region	Modality
*f*_1_	Root Mean Square	Intensity	Enhanced	T1c
*f*_2_	GLCM_Contrast	Texture	Enhanced	T1c
*f*_3_	GLRLM_Low Gray‐level Run Emphasis	Texture	Core	T1
*f*_4_	GLRLM_Short Run Low Gray‐level Emphasis	Texture	Edema	FLAIR
*f*_5_	GLSZM_Gray‐level Nonuniformity	Texture	Edema	T2
*f*_6_	GLSZM_Large Zone High Gray‐level Emphasis	Texture	Enhanced	T1c
*f*_7_	GLSZM_Zone‐Size Variance	Texture	Whole Tumor	T2
*f*_8_	NGTDM_Business	Texture	Non‐enhanced	T1

GLCM, gray‐level co‐occurrence matrix; GLRLM, gray‐level run length matrix; GLSZM, gray‐level size zone matrix; NGTDM, neighborhood gray‐tone difference matrix.

**Table 5:** A summary of the selected features used for building the single‐region models, the tumor‐core model, the whole‐tumor model, and the combined model

Models	Selected features
Enhanced	Root Mean Square_Intensity_T1c, Energy_Intensity_T2, GLCM_Contrast_T1c, GLCM_Informational Measure of Correlation 1_T1c, GLCM_Homogeneity 1_T1c, GLCM_Inverse Difference Moment Normalized_T2, GLRLM_Gray‐level Variance_FLAIR, GLSZM_Large Zone High Gray‐level Emphasis_T1c
Non‐enhanced	Energy_Intensity_T2, GLCM_Contrast_FLAIR, GLCM_Energy_T1c, GLRLM_Low Gray‐level Run Emphasis_T1c, GLRLM_Run‐length Nonuniformity_T2, GLSZM_Zone‐Size Variance, NGTDM_Business_T1
Necrosis	Skewness_Intensity_T2, Energy_Intensity_T1c, Root Mean Square_Intensity_T1c, GLCM_Informational Measure of Correlation 1_T1c, GLCM_Informational Measure of Correlation 2_T1c, GLRLM_Gray‐level Variance_T1
Edema	Energy_Intensity_T2, GLCM_Difference Entropy_FLAIR, GLCM_Informational Measure of Correlation 1_FLAIR, GLRLM_Low Gray‐level Run Emphasis_T2, GLRLM_Short Run Low Gray‐level Emphasis_FLAIR, GLRLM_Gray‐level Nonuniformity_T2, GLSZM_Gray‐level Nonuniformity_T2, GLSZM_Zone‐Size Variance_FLAIR
Tumor core	Uniformity_Intensity_T1c, Energy_Intensity_T1c, GLCM_Dissimilarity_FLAIR, GLCM_Inverse Difference Moment Normalized_T2, GLRLM_Low Gray‐Level Run Emphasis_T1c, GLRLM_Gray‐Level Nonuniformity_T2, GLSZM_Nonuniformity_FLAR, NGTDM_Business_T1
Whole tumor	GLCM_Contrast_T1c, GLCM_Correlation_T1, GLCM_Information Measure of Correlation 1_FLAR, GLCM_Inverse Difference Moment Normalized_T2, GLRLM_Gray‐level Nonuniformity_T1c, GLRLM_Short Run Low Gray‐Level Emphasis_T2, GLSZM_Small Zone Low Gray‐Level Emphsis_FLAIR
Combined	Age, GLCM_Contrast_Enhanced_T1c, GLRLM_Low Gray‐level Run Emphasis_Core_T1, GLRLM_Short Run Low Gray‐level Emphasis_Edema_FLAIR, GLSZM_Gray‐level Nonuniformity_Edema_T2, GLSZM_Zone‐Size Variance_WholeTumor_T2, NGTDM_Business_Nonenhanced_T1

GLCM, gray‐level co‐occurrence matrix; GLRLM, gray‐level run length matrix; GLSZM, gray‐level size zone matrix; NGTDM, neighborhood gray‐tone difference matrix.

**Table 6:** A performance summary of the single‐region radiomics models, multiregional radiomics models, clinical model, and combined model

Models	Primary cohort						Independent validation cohort					
	ACC	SEN	SPE	PRE	AUC	F1	ACC	SEN	SPE	PRE	AUC	F1
Enhance	0.97	0.95	0.98	0.97	0.97	0.96	0.95	0.60	0.99	0.86	0.80	0.71
Non‐enhance	0.95	0.91	0.97	0.94	0.96	0.93	0.88	0.70	0.98	0.78	0.88	0.74
Necrosis	0.95	0.91	0.96	0.93	0.94	0.92	0.95	0.60	0.99	0.86	0.80	0.71
Edema	0.98	0.94	0.99	0.99	0.95	0.97	0.96	0.60	0.99	0.99	0.84	0.75
Tumor core	0.97	0.91	0.99	0.99	0.95	0.95	0.96	0.60	0.99	0.99	0.86	0.75
Whole tumor	0.98	0.96	0.99	0.99	0.96	0.98	0.96	0.60	0.99	0.99	0.88	0.75
**All‐region**	0.97	0.94	0.99	0.98	0.97	0.96	**0.96**	**0.70**	**0.99**	**0.88**	**0.90**	**0.78**
Clinical	0.84	0.80	0.86	0.87	0.84	0.75	0.79	0.72	0.85	0.85	0.80	0.71
**Combined**	0.94	0.91	0.95	0.89	0.94	0.90	**0.97**	**0.80**	**0.99**	**0.89**	**0.96**	**0.84**

The all‐region model achieved an improved overall performance compared with single‐region model in terms of accuracy and ACU, while the combined model achieved the best overall performance (in bold).

ACC, accuracy; AUC, area under the receiver operating characteristic curve; PRE, precision; SEN, sensitivity; SPE, specificity.

## 4 DISCUSSION

The major finding of this study was that radiomics‐based classification with a minimal set of multiregional MRI features allowed for prediction of IDH1 mutation in GBM with high accuracy. The all‐region model outperformed the single‐region models or predictive model built with clinical factors alone. Recent studies have revealed the multiregional and microenvironmental heterogeneity in GBM.^8^, ^9^ It highlights the value of multiregional imaging features in spatially distinct habitats, some of which harbor heterogeneous tumor populations.^19^, ^33^ The prognostic value of multiregional radiomics features has been recognized in GBM.^20^, ^22^ However, many existing studies relating imaging features with IDH mutation lack regional analysis, expressing imaging phenotype as a single value from a single tumorous region.^13^, ^14^, ^15^ The work in ref. [17] predicted IDH1/2 genotypes with multiregional radiomics features in a combined cohort of grade III and IV gliomas. The work in ref. [34] investigated six regional imaging parameters to estimate IDH1 mutation status in GBM. To our knowledge, multiregional radiomics model for prediction IDH1 status in GBM has not been evaluated.

Several previous studies predicted IDH1 mutations with visually‐assessed morphological features, volumetric variables, and blood flow parameters.^34^, ^35^ These features may not fully characterize the imaging phenotypes, thusly limited the potential of imaging genomics models. The studies in ref. [13] and [17] constructed their machine learning‐based radiomics models using 110 and 386 radiomics features for IDH status prediction, respectively. They could have risk of overfitting on future observations as the feature number was significantly high (even higher than the numbers of patients used for training the models). As a well‐recognized principle in machine learning field, a small (possibly minimal) feature set can increase both the model generalizability and interpretability. The work in ref. [14] built a more compact model for prediction of IDH1 status in low‐grade glioma with three single‐region features. In the above studies, the AUCs ranged from 0.79 to 0.92 while the accuracies ranged from 80% to 90%. Our 8‐feature all‐region model and combined model achieved higher accuracy (96% and 97%) and AUC (0.90 and 0.96) in a multicenter independent validation cohort. Our study was based on 1614 features derived from multiple 3D tumor subregions in multiparametric MRI, allowing for a more comprehensive characterization of the radiological heterogeneity. To improve both generalizability and interpretability, we selected a minimal set of the most relevant multiregional features by means of the Boruta algorithm.^30^ To overcome the inherent intensity variability across multicenter MRI acquisitions, we normalized the image intensity via an effective landmark‐based mapping approach.^24^ To tackle the imbalanced learning problems caused by the low incidence of GBM IDH1 mutations (12%^2^), we resampled the data by using a minority class oversampling method.^28^ These efforts may offer potential to improve the prediction performance.

Our results show that among all single‐region models the model built from edema area achieved the highest accuracy (0.96) and F1‐score (0.75), while the model built of non‐enhancement area reached the highest AUC (0.88). It indicated that the imaging phenotypes within distinct tumor subregions may contribute differently to the outcome. The work in ref. [33] has demonstrated that tumor heterogeneity is not limited within the solid core margins but also involves the edema area. A recent study in ref. [36] has demonstrated that radiomics features from the edema predicted survival better than from enhancement area and necrosis. The work in ref. [37] showed that a greater proportion of non‐enhancing area is associated with IDH1 mutation in GBM. Interpretation of the different predictive power of regional imaging features remains challenging. We tried to understand the results from the basic radiomics hypothesis—imaging phenotypes could be the expression of underlying biological or genetic heterogeneity.^10^, ^11^ Genetic heterogeneity is typically due to random mutations and is the result of a predictable Darwin selection of successful cellular adaptive strategies to local microenvironmental conditions.^38^ Multiple studies contend that spatially distinct subregions harbor heterogenous subclones, each with a distinct set of microenvironmental selection forces.^19^ Based on the hypothesis, imaging features characterizing regional variations in blood flow, cell density, and necrosis could identify regional variations in microenvironmental selection forces. The feature maps gave an example of how multiregional imaging features radiologically quantified the multiregional variations.

Previous genomics study has revealed that IDH1 mutations are much more common in younger GBM patients.^3^ A high‐resolution gene expression analysis has demonstrated that pediatric and adult high‐grade glioma are clearly distinguished by the absence of IDH1 hotspot mutations.^39^ Our result showed that age was selected by the combined model as a predictor of IDH1 mutations, reflecting the previous observations. Combing age with multiregional imaging features resulted in a better prediction performance than using imaging features or clinical factors alone. Similar results have been observed in ref. [17], confirming the advantage of combining radiomics features with age for predicting GBM IDH1 mutation status.

There were several limitations to our study. First, although this study was based on multicenter cohorts, larger prospective cohorts from more institutes should be involved to demonstrate the potential clinical utility of our model. Larger multicenter cohorts also have great potential to improve the performance of the machined learning‐based radiomics approach, especially for this imbalanced learning problem. Second, our model made use of four MR modalities. Recent studies have shown that MR spectroscopy, diffusion‐weighted imaging (DWI), diffusion tensor imaging (DTI), and arterial spin labeling (ASL) perfusion MR imaging are promising in predicting IDH1 mutation status in glioma.^34^, ^40^, ^41^, ^42^ Incorporating features calculated from these new modalities may potentially improve the performance of our multiregional model.

In conclusion, the radiomics‐based model with a minimal set of multiregional features from multiparametric MRI has the potential to noninvasively detect the IDH1 status in preoperative GBM patients. The multiregional radiomics models performed better than the single‐region models, while combining age with multiregional features achieved the best prediction performance.
